# Honey Bee Products: Preclinical and Clinical Studies of Their Anti-inflammatory and Immunomodulatory Properties

**DOI:** 10.3389/fnut.2021.761267

**Published:** 2022-01-03

**Authors:** Hesham R. El-Seedi, Nehal Eid, Aida A. Abd El-Wahed, Mostafa E. Rateb, Hanan S. Afifi, Ahmed F. Algethami, Chao Zhao, Yahya Al Naggar, Sultan M. Alsharif, Haroon Elrasheid Tahir, Baojun Xu, Kai Wang, Shaden A. M. Khalifa

**Affiliations:** ^1^Pharmacognosy Group, Department of Pharmaceutical Biosciences, Biomedical Centre, Uppsala University, Uppsala, Sweden; ^2^International Research Center for Food Nutrition and Safety, Jiangsu University, Zhenjiang, China; ^3^International Joint Research Laboratory of Intelligent Agriculture and Agri-Products Processing, Jiangsu Education Department, Jiangsu University, Zhenjiang, China; ^4^Department of Chemistry, Faculty of Science, Menoufia University, Shebeen El-Kom, Egypt; ^5^Department of Bee Research, Plant Protection Research Institute, Agricultural Research Centre, Giza, Egypt; ^6^School of Computing, Engineering & Physical Sciences, University of the West of Scotland, Paisley, United Kingdom; ^7^Food Research Section, R&D Division, Abu Dhabi Agriculture and Food Safety Authority (ADAFSA), Abu Dhabi, United Arab Emirates; ^8^Alnahalaljwal Foundation Saudi Arabia, Mecca, Saudi Arabia; ^9^College of Food Science, Fujian Agriculture and Forestry University, Fuzhou, China; ^10^General Zoology Group, Institute for Biology, Martin Luther University Halle-Wittenberg, Halle, Germany; ^11^Zoology Department, Faculty of Science, Tanta University, Tanta, Egypt; ^12^Biology Department, Faculty of Science, Taibah University, Medina, Saudi Arabia; ^13^School of Food and Biological Engineering, Jiangsu University, Zhenjiang, China; ^14^Programme of Food Science and Technology, BNU-HKBU United International College, Zhuhai, China; ^15^Institute of Apicultural Research, Chinese Academy of Agricultural Sciences, Beijing, China; ^16^Department of Molecular Biosciences, The Wenner-Gren Institute, Stockholm University, Stockholm, Sweden

**Keywords:** bee products, inflammation, diabetes, hypertension, cancer, preclinical and clinical studies, safety

## Abstract

Inflammation is a defense process triggered when the body faces assaults from pathogens, toxic substances, microbial infections, or when tissue is damaged. Immune and inflammatory disorders are common pathogenic pathways that lead to the progress of various chronic diseases, such as cancer and diabetes. The overproduction of cytokines, such as interleukin (IL)-1β, IL-6, and tumor necrosis factor-α, is an essential parameter in the clinical diagnosis of auto-inflammatory diseases. In this review, the effects of bee products have on inflammatory and autoimmune diseases are discussed with respect to the current literature. The databases of Google Scholar, PubMed, Science Direct, Sci-Finder and clinical trials were screened using different combinations of the following terms: “immunomodulatory”, “anti-inflammatory”, “bee products”, “honey”, “propolis”, “royal jelly”, “bee venom”, “bee pollen”, “bee bread”, “preclinical trials”, “clinical trials”, and “safety”. Honey bee products, including propolis, royal jelly, honey, bee venom, and bee pollen, or their bioactive chemical constituents like polyphenols, demonstrate interesting therapeutic potential in the regulation of inflammatory mediator production as per the increase of TNF-α, IL-1β, IL-6, Il-2, and Il-7, and the decrease of reactive oxygen species (ROS) production. Additionally, improvement in the immune response via activation of B and T lymphocyte cells, both in *in vitro, in vivo* and in clinical studies was reported. Thus, the biological properties of bee products as anti-inflammatory, immune protective, antioxidant, anti-apoptotic, and antimicrobial agents have prompted further clinical investigation.

## Graphical Abstract

The anti-inflammatory and immunomodulatory possible mechanisms of action of bee products suggested by preclinical and clinical studies. 
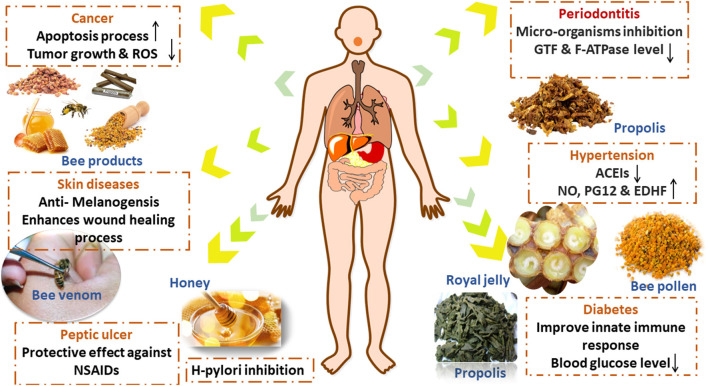

## Introduction

Inflammation is a defense mechanism that the immune system employs as a response to foreign stimuli, such as pathogens, toxic substances, bacterial infections, and irradiation, to repair tissue damage and return it to a state of hemostasis. Redness, swelling, heat, and pain are characteristic inflammatory processes involving various immune cells, such as B and T lymphocytes, macrophages, monocytes, basophils and neutrophils, mast and dendritic cells (1). The inflammatory response is mostly dependent on the nature of the stimuli; however, they share common mechanisms, such as the recognition of cell surface receptors, called pattern-recognition receptors, present on foreign pathogens or intracellular signaling that occurs because of damaged cells or tissues. The initiation of the inflammatory process activates different types of signaling, such as mitogen-activated protein kinase (MAPK), signal transducer, activator of transcription (STAT), nuclear factor kappa-B (NF-κB), and Janus kinase (JAK) pathways, leading to the release of inflammatory mediators, e.g., chemokines and cytokines, and the recruiting of inflammatory and immune cells. The NF-κB signaling pathway initiates the regulation of inflammatory cytokine production and the migration of inflammatory cells to injured tissues (2). Similarly, the MAPK pathway induces a cellular response to pathogens via protein kinase cascades that aid the regulation of the cell proliferation (3). Also, the JAK–STAT pathway promotes the release of various inflammatory cytokines, growth factors, and interferons (2). Cytokines are proteins with a molecular weight of <40 kDa that are released by immune cells, such as macrophages, monocytes, and lymphocytes, and are considered to be modulatory actors in acute and chronic inflammation (4). Uncontrolled acute inflammation, an overlap in the activation of intracellular signaling pathways, and the release of inflammatory mediators result in the onset of chronic inflammatory diseases, such as diabetes and cancer (2), peptic ulcers (5), cardiovascular, and periodontal diseases (6).

**Figure 1:**
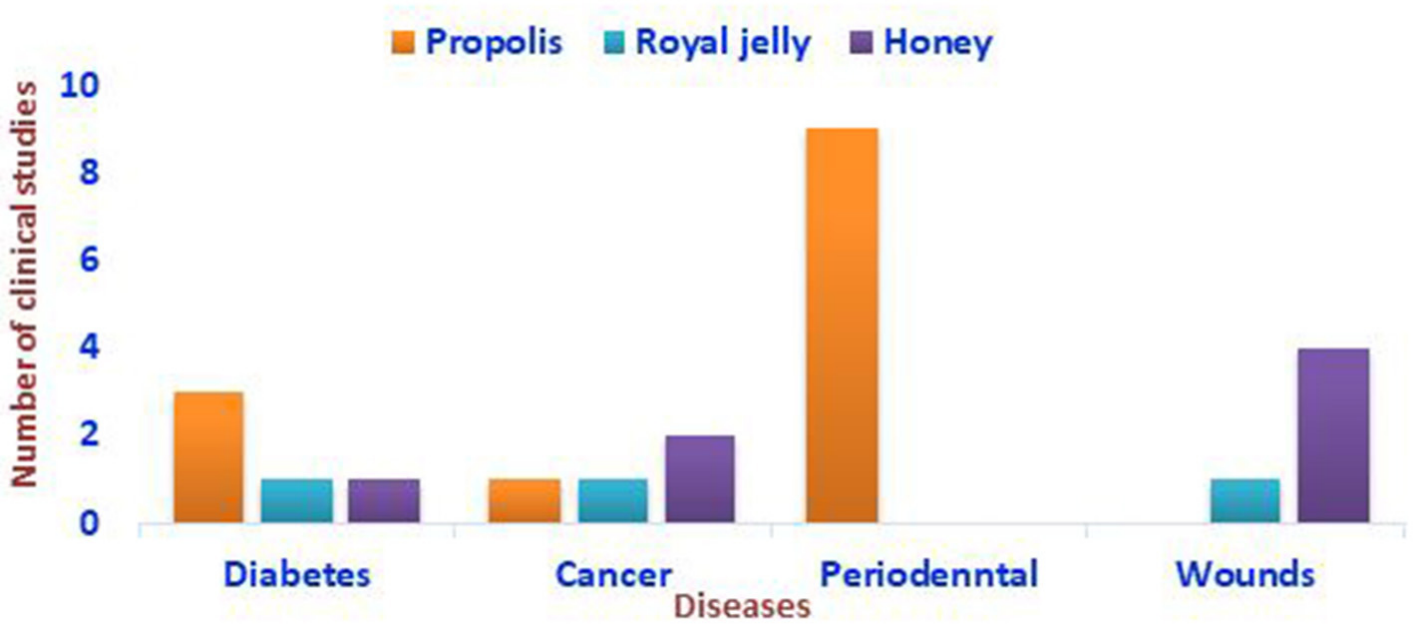
Representation of the clinical studies conducted on the impacts of bee products against some autoimmune diseases based on the literature screening.

Honey, propolis, royal jelly, bee pollen, bee bread, venom, and wax are all products produced by bees. The health, nutritive, and medicinal values of bee products have been demonstrated by the ancient Egyptians, Greeks, and Chinese (7). Bee products have long been used as health-promoting nutritional additives (8). Bee products have diverse biological properties such as antimicrobial, anti-inflammatory, anticancer, and antioxidant activities (9, 10). Proteins, peptides, minerals, flavonoids, terpenes, fatty acids, and phenolic compounds are among the physiologically active components that exist in bee products (8, 11, 12). Propolis is a thick, resinous, lipophilic bee product known as “Bee glue & Russian Penicillin.” Propolis is a sticky substance that honeybees gather by mixing their own waxes with resinous sap obtained from the bark and leaf-buds of certain trees and other flowering plants, to use it in their nests. Chemically, it is composed of 50% plant resins, 30% waxes, 10% essential and aromatic oils, 5% pollens, and 5% other organic substances (1). It is known to have antibacterial, antiviral, anticancer, and anti-inflammatory effects. It is also reportedly the most potent antioxidant bee product (13–16). The propolis ethanolic extract (EEP) and its phenolic components have been shown to induce tumor necrosis factor-related apoptosis-inducing ligand (TRAIL)-mediated apoptosis in cancer cells (17). It is also worth mentioning that bee venom and melittin, a major component of bee venom, have demonstrated anti-inflammatory and immunomodulatory activities. Bee venom is a natural toxin that is secreted by the poison and accessory gland of honeybee workers, located in the abdominal cavity. It is then stored in the venom reservoir and plays a significant role in the defense strategy of bee colonies. Bee venom has anti-inflammatory, radio-protective, and potent antibacterial effects. It also has demonstrated effectiveness in treating certain pathological conditions, such as rheumatism, and cancer, as it can kill cancer cells (18, 19). Bee venom contains biologically active components such as melittin, apamin, adolapin, secapin, tertiapin, mast cell-degranulating phospholipase A2, and hyaluronidase. Histamine, epinephrine, and macromolecules (such as lipids, carbohydrates, and free amino acids) are also among the bioactive elements (2). Melittin combined with nanographene oxide showed anticancer activity toward MCF-7 and MDA-MB-231 breast cancer cells compared with melittin alone, and it also helped to reduce the unselective cytotoxicity toward normal cells (20). Honeybee pollen is a mixture of pollen and flower nectar that honeybees collect and mix as food supply for the hive. Proteins, amino acids, saccharides, vitamins, and minerals abound in it. As a result, it is commonly considered a “super food,” one of the most nutrient-dense natural items, and is commercially available as a food supplement (21). Honeybees primarily use royal jelly to feed the queen and young larvae. It is, nevertheless, one of the functional bee products, that has been manufactured for commercial usage in many nations, particularly in the fields of dietary products and cosmetics. Royal jelly is an acidic emulsion made up of fat, protein, and carbohydrates suspended in 60 to 70% water. The sugars glucose and fructose account for 90% of the total sugar content of royal jelly. The lipid content of royal jelly is unusual component, consisting primarily of short-chain fatty acids, mostly hydroxyl and carboxylic acids, which are distinct from other organic acids found in animals and plants (8–12). Royal jelly has antimicrobial, anti-inflammatory, immunomodulatory, and anti-diabetic properties, making it a commercially and medicinally valuable material (22, 23). The effectiveness of bee products, such as bee venom, royal jelly, propolis, bee pollen, and honey, in treating inflammatory and autoimmune diseases, have been documented in various reports (24–27). However, there are still few reports of clinical studies on bee products (Figure 1). This is due to the incidence of asthma and anaphylaxis, in some instances upon the consumption of royal jelly, bee venom, and bee pollen, which somewhat hinders their use for some individuals despite their medicinal and health benefits (28–30).

This review comprehensively outlines the preclinical and clinical studies that have been carried out to date on bee products and their bioactive ingredients, emphasizing their role in treating the immune and inflammatory disorders.

## Immunomodulatory and Anti-Inflammatory Properties of Bee Products

Inflammation is a common underlying cause of serious diseases, as it initiates a pathogenic cascade of the failure of metabolic pathways, tissue damage, and even necrosis and apoptosis. Inflammatory diseases are diagnosed after an increase in interleukin (IL)-1β, IL-6, and tumor necrosis factor-α (TNF-α) cytokines (2). The *in vitro* and *in vivo* investigations have shown that bee products and bee products derived active constituents show potent anti-inflammatory and anti-autoimmune properties. The 8–12 C free fatty acids in royal jelly, such as 10-hydroxydecenoic acid (10-HDA), have modulated the immunological response in a concentration-dependent manner. In an *in vitro* study, 10-HDA at a dose of 500 mM inhibited the maturation of lipopolysaccharide (LPS)-stimulated human monocyte-derived dendritic cells (Mo-DCs) and the production of IL-8, IL-12, and TNF-cytokines, as well as down-regulating both Th1 and Th2 immune responses and decreasing the number of T helpers (Th1 and Th2) and Mo-DCs. A higher concentration of 10-HDA of more than 750–1,000 μM induced Mo-CD cell apoptosis (31). *In vitro* and *in vivo* studies, bee venom and melittin present a potential means to stop overactive immune responses and inflammatory disorders; however, the cytotoxic effect of melittin needs to be taken into account (32). *Porphyromonas gingivalis* and its lipopolysaccharides (PgLPS) are known pathogens that are involved in periodontal disease. Bee venom shows an inhibitory effect on the pro-inflammatory cytokines IFN-γ, IL-1β, IL-6, IL-8, and TNF-α by suppressing the NF-κB and AP1 pathways induced by PgLPS in the human keratinocyte cell line (HaCaT cells, *in vitro*) (33). Moreover, a polysaccharide of Chinese wolfberry bee pollen exhibited an immunomodulatory response toward RAW264.7 cells (*in vitro*) by decreasing the levels of IL-1, IL-6, TNF-α, and nitric oxide (NO) secretion (34). Topical application of propolis improved the wound healing process of a streptozotocin (STZ)-induced wound in a type I diabetes mellitus (DM) mouse model, which was attributed to a decrease in pro-inflammatory cytokines such as IL-6, IL-1B, and TNF-α, as well as an increase in collagen formation via the transforming growth factor-1 (Smad2 and Smad3) signaling pathways (35). Overall, the anti-inflammatory and immunomodulatory effects of bee products mainly venom, royal jelly, and propolis are linked to the secretion of pro-inflammatory cytokines such as IFN-, IL-1, IL-6, IL-8, and TNF-α, as well as the regulation of intra signaling pathways and modulation of B and T lymphocyte cell function.

## Preclinical and Clinical Studies on Bee Products

### Diabetes

#### Preclinical Studies

Diabetes (DM) is one of the chronic diseases that develop due to severe inflammation and manifests in an increase in pro-inflammatory cytokines and chemotaxis mediated by B and T lymphocytes, such as IL-1β and TNF-α, leading to insulin resistance (36). In mice model, the immunomodulatory activity of propolis was found to improve the innate immune response. It induces the up-regulation of Toll-like receptors 2 (TLR-2) and TLR-4, which leads to the activation of lymphocytes, and CCL21- and CXCL12-mediated chemotaxis by B and T lymphocytes. Also, propolis helps to restore the levels of IL-1β, IL-6, Il-2, Il-7, and TNF-α (37, 38). In diabetic male rats model, propolis decreases serum immunoglobulin (IgG, IgA, and IgE), that controls B and T lymphocyte proliferation (39). The peptic bee pollen polysaccharide (RBPP-P) of *Rosa rugosa* shows a regulatory effect toward certain pro-inflammatory cytokines that raise insulin resistance, e.g. TNF-α and IL-6. Treatment with RBPP-P decreases TNF-α and IL-6 levels, resulting in less insulin resistance observed in the tissues of mice fed with a high-fat diet (40).

Furthermore, DM is characterized by hyperglycemia, glycosuria, and dyslipidemia. Dyslipidemia and hyperglycemia induce the propagation of reactive oxygen species (ROS), which lead to cellular dysfunction and oxidative injuries, especially to the heart, kidney, and liver tissues (41). Propolis, royal jelly, and bee pollen act against the oxidative stress that arises in different diseases, e.g., DM (42). Propolis has anti-hyperglycemia, dyslipidemia, and antioxidant properties, which could be attributed to its polyphenolic content, e.g., α-amylase, α-glucosidase, and caffeic acid phenethyl ester (CAPE) (43, 44). Propolis improves lipid profile metabolism and decreases the ROS levels in blood, liver, and lymph nodes (45). Intraperitoneal administration of water-soluble-derivative of propolis (WSDP) or (–)-epigallocatechin gallate (EGCG) (50 mg/kg/day) in alloxan-induced diabetic mice for 7 days lead to reduce in lipid peroxidation in liver, kidney, brain tissues, and reduce DNA damage in peripheral lymphocytes of diabetic mice (46). WSDP and ethanolic propolis extract increased the animal body weight and the life span of treated mice vs. the untreated mice mediated by the improvement of the fatty acid metabolism impairment (47). In addition to this, propolis significantly controls body weight and bone homeostasis (48). An ethanolic extract of propolis has reduced hyperglycemia and protected the pancreatic and renal tissue of STZ-induced diabetic rats that received 30 or 15% propolis extract at a dosage of 50 ml/kg for 4 weeks. Blood sugar levels decreased from 393 ± 192.7 to 154 ± 28.0 mg/dl and from 386 ± 141.1 to 331.5 ± 123.74 mg/dl, was observed in the diabetic groups treated with 30 and 15% propolis, respectively. An improvement at the pancreatic, hepatic, and renal tissue levels was seen in both groups treated with propolis compared with the control group. The IC_50_ results for α-amylase (0.62 ± 0.00 μg/ml) and α-glucosidase (40.40 ± 0.09 μg/ml) whereas, CAPE and chrysin were the dominant phenolics (43). Bee pollen or date palm pollen suspension (100 mg/kg) administration for four weeks has shown to reduce body, testis, and pancreas weight and insulin resistance in STZ-induced diabetic Wistar male rats. The protective effect of bee pollen was found to be associated with a decrease in NO and LPx-biomarkers of oxidative stress and increase in glutathione (GSH), GSH-S-transferase, GSH peroxidase, and superoxide dismutase (SOD) antioxidant defense markers (49).

#### Clinical Studies

**Figure 2:**
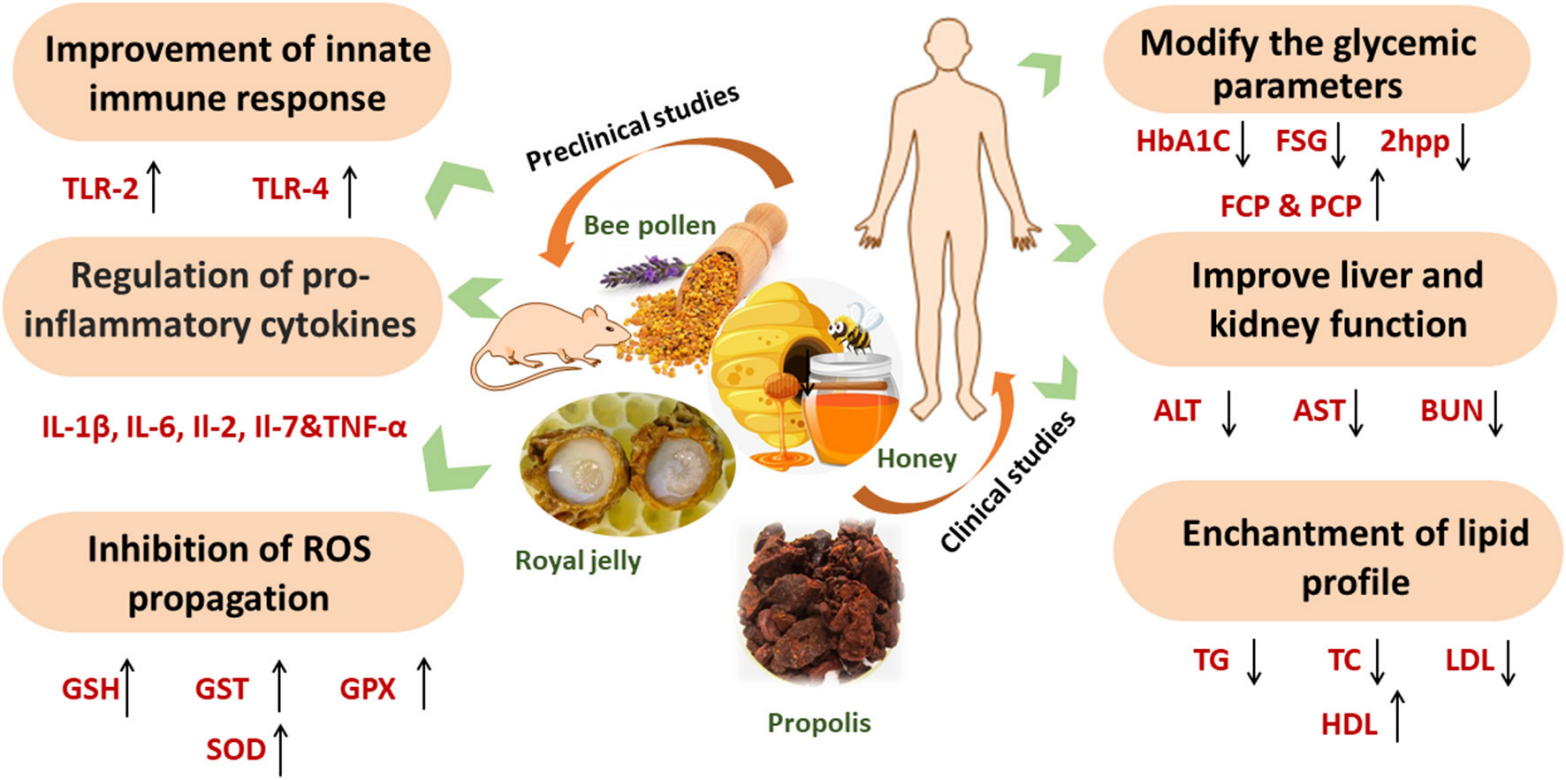
The therapeutic potential of bee products in diabetes.

Propolis, royal jelly, and honey have been the subject of various clinical studies, as shown from the data presented in Table 1. Propolis administrated at different doses (300 and 400 mg) has shown to aid in the modulation of the glycemic mediators fasting serum glucose (FSG) and glycosylated hemoglobin A1C (HbA1C) in subjects with type 2 DM (T2DM) (51, 52). Meanwhile, the oral administration of encapsulated propolis has enhanced liver and kidney functions as well as lipid profiles by increasing high density lipoprotein (HDL) levels and glucose metabolism and decreasing the levels of HbA1C by 8%, 2-h postprandial (2 hpp), homeostasis model assessment of β-cell function (HOMA-β), and TNF-α as shown in Table 1 (55). The oral administration of encapsulated royal jelly (1,000 mg) three times per day for eight weeks has led to a decrease in glucose (−9.4 ± 13.5 mg/dL vs. 4 ± 8.2 mg/dL in the placebo group) and an increase of serum apolipoprotein A-I (ApoA-I) (34.4 ± 53.3 mg/dL vs. −1.08 ± 32.6 mg/dL in the placebo group) levels and thus diminish the risk of cardiovascular diseases (CVDs) in patients with T2DM (53). Figure 2 outlines the therapeutic potential of bee products in preclinical and clinical studies of diabetes, how different factors influence them, and how they are associated with the underlying pathogenesis.

**Table 1:** The potency of some bee products against diabetes clinically.

**Type of diabetes**	**Recruitment status**	**Bee product/Form/Dose**	**Inclusion criteria**	**Type of the study/Clinical trials phase/No. of participants**	**Mode of action**	**References**
Diabetes Mellitus (T2DM)	Completed	Propolis/Encapsulated/One capsule propolis (400 mg), once daily, for 6 months	Eligible age: 38 to 63 years Eligible gender: Male and female	Interventional/Phase 4/52 P	Modulate glycemic parameter e.g., HbA1C and FPG.	(50)
T2DM	Completed	Propolis/Encapsulated/One capsule propolis (300 mg), 2 times per day, for 12 weeks	Eligible age: 30 to 60 years Eligible gender: Male and female	Interventional/Phase 2/36 P	Modify the glycemic control in patients e.g., HbA1c and FSG.	(51, 52)
T2DM		Royal jelly/Encapsulated/ One capsule of royal jelly (3,000 mg), for 8 weeks	Eligible age: 20 to 60 years Eligible gender: Male and female	Interventional/50 P	Level of serum glucose decreased. Modify the ratio of B (ApoB)/A-I (ApoA-I).	(53)
T1DM	Completed	Honey/Fresh/Honey 0.5 ml/kg, once daily, for 12 weeks	Eligible age: 4 to 18 years Eligible gender: Male and female	Interventional/Phase 2/20 P	Improvement to lipid profile and hyperglycemia (FSG, PSG, TC, TG and LDL). Increasing in postprandial C-peptide (PCP) and FCP which associated with promoting of beta cells for insulin secretion.	(54)
T2DM	Not reported	Propolis/Encapsulated/One capsule of propolis (500 mg)/(1000 mg/day), for 90 days	Eligible: 35 to 85 years Eligible gender: Male and female	Interventional/100 P	Improvement to glucose metabolism by decreasing of HbA1C, 2hpp and HOMA-β Reducing TNF-α level. Reducing liver and renal dysfunction by the decrease of ALT AST, and BUN. Enhances lipid profile by significant increase of HDL.	(55, 56)
T1DM	Not reported	Honey/Raw/0.5 mL/kg per day for 12 weeks	Eligible: 4–18 years Eligible gender: Male and female	Interventional/ Not reported/20 P	Consumption of honey has positive effects on hypoglycemia and lipid profile metabolism by decreasing SSFT, FSG, TC, TG, and LDL. Significant increases in FCP and PCP.	(57)

### Cancer

#### Preclinical Studies

In the 19^th^ century, the detection of leukocytes in tumor cells was the first indication of the link between cancer and inflammation since 20% of cancers could be attributed to inflammation. Inflammation is involved in the different steps of tumor development, e.g., initiation, promotion, malignancy, invasion, and metastasis (58). The term chemoprevention describes the inhibition of the processes of mutagenesis, carcinogenesis, proliferation, and the progression of tumor cells using natural or synthetic compounds (59, 60).

Honey and its polyphenolic compounds, e.g. pinobanksin, pinocembrin, luteolin, galangin, chrysin, and quercetin (3,3′,4′,5,7-pentahydroxyflavanone), shown to have anti-inflammatory, immunomodulatory, anti-proliferative, and anti-metastatic properties. They were found to induce apoptosis and showed antioxidative activity toward several cancer types (61). Tyrosine-phosphorylated signal transducer and transcription three activator (p-STAT3) is a major player in lung and breast cancer (62). Manuka honey (MH) has shown to have an inhibitory effect on p-STAT3 in the triple-negative breast cancer cell line MDA-MB-231 and non-small cell lung cancer cell line A549. MH targets the IL-6 receptor α chain (IL-6Rα), preventing its binding to IL-6 by ~60%, thus decreasing the level of glycoprotein (gp130) and tyrosine-phosphorylated JAK2, which are important components of IL-6Rα. Galangin, chrysin, quercetin, and luteolin are the major flavonoid compounds in MH that can inhibit the binding of IL-6Rα and IL-6 by 34.3, 31.8, 29.2, and 22.4%, respectively, at a concentration of 50 μM (63).

Propolis containing mainly polyphenolic compounds has exhibited chemoprevention and tumor growth inhibition abilities, as well as antioxidant, pro-oxidant, and immunoprotective properties (64–66). These compounds have been reported to induce apoptosis, inhibit matrix metalloproteinase, regulate cell proliferation by promoting cytotoxicity, modulate gene methylation (67), and exhibit anti-inflammatory and anti-angiogenic activities toward urothelial and bladder cancer (68). WSDP and its polyphenolic compounds caffeic acid (CA) and CAPE show immunomodulatory properties and control the growth and spread of tumors. The administration of WSDP, CA, and CAPE at dose 50 or 150 mg/kg via intravenous injection into mice with mammary carcinoma was shown to decrease the number of colonies. In contrast, subcutaneous administration inhibited tumor growth and increased the survival of the mice. In the proposed mechanism, the phenolic compounds initiate the anti-tumor activity of macrophages, which help to block the metastasis process and increase hydrogen peroxide production, which modulates lymphocyte function. This is in agreement with the inhibition response of spleen cells toward the administration of polyclonal mitogens (phytohemagglutinin (PHA), concanavalin A (ConA), pokeweed mitogen (PWM), and LPS in mice treated with CA (65). WSDP inhibits IL-1, which is associated with B and T cell proliferation (65).

**Figure 3:**
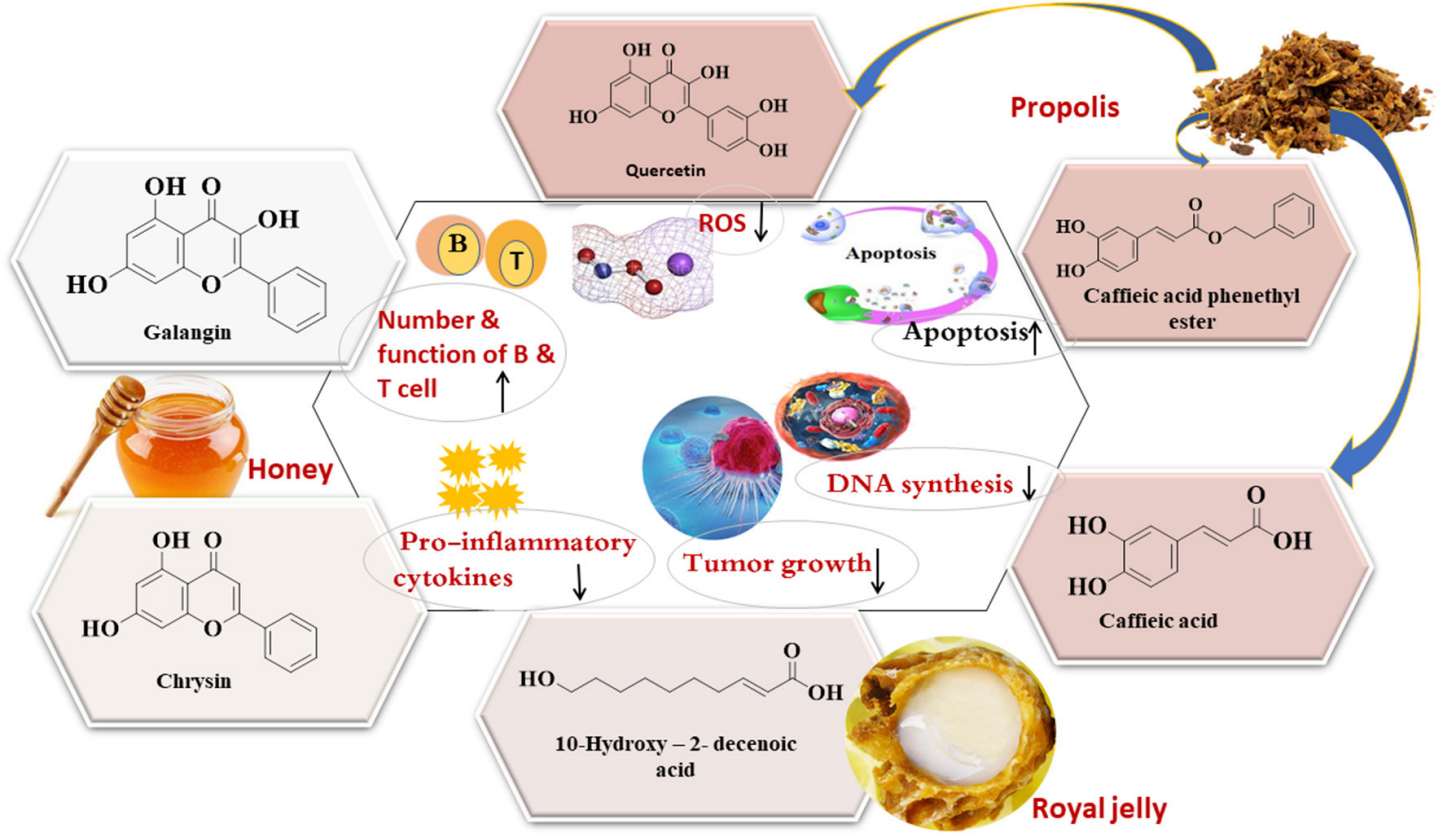
Representative bioactive compounds from bee products as anti-cancer agents.

Also, 10-HDA in royal jelly exhibits anti-inflammatory properties toward human colon adenocarcinoma cells. 10-HDA inhibits the production of pro-inflammatory cytokines, TNF-α, IL-1β, and IL-8 in WiDR human adenocarcinoma cell (BCRC 60157). Receptor antagonist cytokine (LI-1ra) production induced by 10-HDA at doses of 0.1–3 mM helps to reduce tumor growth and stop the production of IL-8 and IL-1, which are causative factors in the development of some cancers, including pancreatic and colon carcinoma (69). CAPE causes distinctive changes to breast cancer stem cells (bCSCs) as it halts the generation of malignant cells by suppressing their self-generation and CD44 clonal expansion. Additionally, the results suggest that MDA-231-derived bCSCs treated with CAPE are less susceptible to malignant infections (70). TRAIL is an effective endogenous anticancer agent that promotes apoptosis, especially of tumor cells. An ethanolic extract of green propolis promote TRAIL-mediated apoptosis in LNCaP prostate cancer cells via the modulation of the intrinsic and extrinsic apoptotic pathways and alteration of the NF-κB activity. Brazilian green propolis able to enhance TRAIL-mediated apoptosis, which could be one of the mechanisms responsible for its cancer-preventive properties. The major components of Brazilian green propolis determined by high-performance liquid chromatography (HPLC) including; quercetin, artepillin C, kaempferol (KAE), and *p*-coumaric acid. Brazilian EEP at 50 μg/mL and its components exhibit better free radical scavenging than the other compounds in the propolis extract at 50 μg/mL. Quercetin was the most bioactive compound that induces cell death and apoptosis toward LNCaP (71). Collectively, galangin, chrysin, CA, CAPE, and 10-HDA are the common bioactive components of bee products that show anticancer activity (Figure 3).

Despite its potential anticancer activity, clinical studies/trials on propolis are limited due to its poor physicochemical properties and oral bioavailability. Thus, the encapsulation of propolis extract in nanoparticle systems has been suggested. Through a well-designed polymeric architecture, propolis encapsulation in nano-in-microparticles (NIMs) enhances bioavailability, therapeutic efficiency, and controlled drug release. Nano spray drying is a method for producing polymeric nanoparticles, microparticles, and NIMs that contain a variety of bioactive compounds and drugs. The prepared spray-dried NIMs of propolis extract have been supplemented orally for the treatment of colon and liver cancer and were found to promote the cytotoxicity of human liver (HepG2) and colon (HCT-116) carcinoma cell lines (67, 72).

The application of propolis and lycopene extract nanoemulsions is safe and has been shown to have a highly protective effect on skin cells against UV irradiation, which induces different skin cancers via erythema and pigmentation. Nanoemulsions have been widely applied in the delivery of poorly soluble drugs. Nanoemulsions have been shown to increase solubility, kinetic stability, and decrease collagenase activity (73). Also, the trapping of bee venom components in nanoparticles may help to reduce its unselective toxicity and improve its prospects for application as a therapeutic anticancer agent (74).

#### Clinical Studies

Anti-inflammatory and antioxidant properties of honey, propolis, royal jelly, and bee pollen can explain the impact of bee products against different types of cancer. As demonstrated in Table 2, honey and propolis are used to treat oral mucositis, lymphoblastic leukemia, and head and neck malignancies, whereas bee pollen is used to treat renal cell carcinoma (75, 77–81). Additionally, propolis and honey reduced the risk of oral mucositis induced by undergoing radiotherapy treatment, as shown in Table 2. The anti-oxidant and anti-inflammatory activities of encapsulated royal jelly (400 mg) were studied and proven protective against the fatigue and anorexia induced by tyrosine kinase inhibitors (TKIs) in patients with renal cell carcinoma (RCC) (76).

**Table 2:** The potency of some bee products against cancer clinically.

**Disease/ condition**	**Recruitment status**	**Bee product/form/dose**	**Inclusion criteria**	**Type of the study/Clinical trials phase/No. of participants**	**Mode of action**	**References**
Radiation-induced mucositis of oral Mucous	Completed	Propolis/Mouth wash/Propolis 15 ml, 3 times per day, for the whole period of radiotherapy	Age: > 15 Year/Gender: Male and female	Interventional/Phase 2/20 p	Not reported	(75)
Renal cell carcinoma (RCC)	Not reported	Royal jelly/Encapsulated/One capsule of royal jelly (400 mg), 4 times per day, for 3 months	Age: > 20 years/Gender: Male and female	Interventional/33 P	Reduce the level of toxicities, decreases fatigue, and anorexia induced by TKIs in RCC.	(76)
Oral mucositis induced by undergoing radiotherapy for head and neck cancers	Not reported	Honey/Raw/Honey, 3 times per day (1 h prior to radiation, and 2 and 6 h after radiation), through the whole period of radiation	Age: > 18 years/Gender: Male and female	Interventional/50 P	Decrease risk of mucositis by reducing ROS level.	(77)
Oral mucositis leukemia	Recruiting	Honey/Raw/Manuka honey, 5 ml for children while 10 ml of honey for adults, dietary supplement	Age: > 5 years/Gender: Male and female/Not accept healthy volunteers	Interventional/Phase 1/60 P	Not reported	(78)
Oral mucositis grades 2 or 3 Lymphoblastic leukemia	Recruiting	Group 1 received: honey (0.5 g to 15 g/kg maximum) Group 1 received: mixture of honey, olive oil–propolis extract and beeswax [4:2:1 (HOPE)] (0.25–5 g/kg maximum)/3 times per day, for 10 days/Topical application	Age: 2–18 years/Gender: Male and Female/ All patients with oral mucositis have chemotherapy-related oral mucositis grades 2 and 3 and have consolidation phase of treatment	Interventional/Phase 2/90 P	Honey caused faster healing in oral mucositis grade 3 more than HOPE.	(68)
Oral mucositis grade 2 or 3		Honey	Age: > 1 year Gender: Male and female	Interventional/Not reported/40 P	Significant reduction in the number of episodes of oral mucositis, bacterial and fungal infections among pediatric cancer patients and this was accompanied by body weight gain in patients.	(79)
Oral mucositis	Recruiting	Propolis and honey/Honey (30 g for three times/day), and 0.5 ml (100 mg/mL), three times/day for 12 weeks	Age: 20–100 Years/Gender: Male and female	Interventional/Not reported/150 P	Not reported	(80)
Breast cancer	Recruiting	Estrogenic pollen extract PCC-100 for 12 weeks	Age: 50–65 Years/ Gender: Female	Interventional/Not reported/300 P	Not reported	(81)

### Hypertension

#### Preclinical Studies

Inflammation is a common predisposing factor of atherosclerosis and hypertension, and consequently, CVDs. Hypertension is a global problem and one of the leading causes of CVDs e.g., cerebral stroke, acute myocardial infarction, and heart failure. The renin–angiotensin system (RAAS) is essential in regulating blood pressure and maintaining inflammatory processes in CVDs. The use of angiotensin-converting enzyme inhibitors (ACEIs), angiotensin receptor blockers, and direct renin inhibitors to inhibit RAAS pharmacological action is effective in the treatment of hypertension (82).

**Figure 4:**
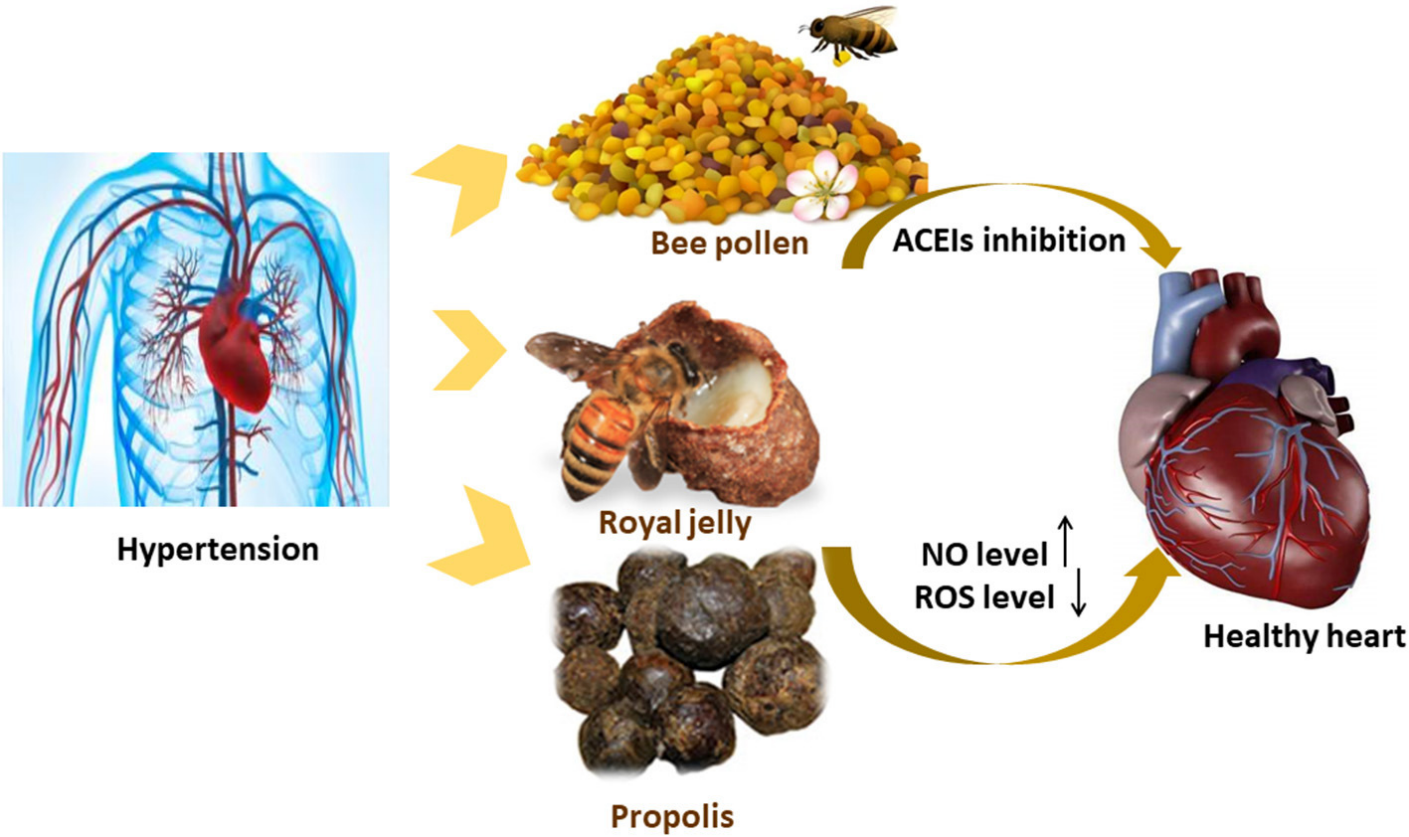
Possible mechanism of actions explaining the anti-hypertensive effect of the bee products.

The antioxidant properties of bee pollen polyphenols have been shown to play an important role in inhibiting the angiotensin-converting enzyme. The supplementation of bee pollen extract (0.1 and 1 g/kg for 16 weeks) decrease the high levels of ACEIs and angiotensin II-induced by high-fat diets in C57BL6 mice, leading to an enhancement in endothelial function due to the modulatory effect it has on RAAS (Figure 4) (83). It has also been noted that protease *N*-treated royal jelly (Pro-RJ) with Ile–Tyr (IY), Val–Tyr (VY), and Ile–Val–Tyr (IVY) shows high inhibitory activity toward ACEIs. The oral administration of Pro-RJ peptide (IY, VY, or IVY) or a peptide mixture (IY, VY, and IVY) for 28 days was shown to yield anti-hypertensive activity. The long-lasting hypotensive activity of royal jelly protein (RJPH) has been attributed to its small molecular weight peptide content and its fast digestion time (84, 85). Major RJPH 1 (MRJP1) is another bioactive component of royal jelly that has shown anti-hypertensive activity. MRJP1 exhibits a high regulatory effect on blood pressure via its action on vascular smooth muscle cells (VSMCs). The MRJP1 shows a protective effect against the contraction, abnormal migration, and proliferation of VSMCs by reducing their movement and energy supply. VSMCs regulate blood pressure as they play the main role in blood vessel contraction and relaxation. Also, the proliferation of VSMCs and the increase in its numbers leads to a reduction in the blood vessel diameter, which in turn affects blood flow (86).

The pathophysiological condition of hypertension is manifested by the decrease in NO production, prostacyclin, and endothelium-derived hyperpolarizing factor, which are endothelium-derived relaxing factors of vascular endothelial cells. Royal jelly has a muscarinic receptor agonist effect that is similar to acetylcholine, which increases NO formation and vasorelaxation via the NO/cGMP pathway and calcium channels. An increase in NO formation promotes vasodilation and hypotension (84). A total of 200 mg/kg of propolis supplementation has reduced oxidative stress in hypertensive rats treated with NO-nitro-L-arginine methyl ester NO synthase inhibitor (Figure 4). ROS causes NO loss by its conversion into a peroxynitrite oxidant. The antioxidative effect of propolis helps in reducing ROS, increasing the levels of NO synthesis, and enhancing the endothelial function (87).

#### Clinical Studies

In clinical studies, it was reported that the utilization of mouthwash containing propolis causes a reduction in the harmful effect of oral bacteria, hence disrupting NO levels and leading to a decrease in blood pressure (88).

### Periodontitis

#### Preclinical Studies

Periodontal disease is a phenomenon where the periodontal tissue accumulates bacterial plaque followed by a secondary immune and inflammatory response (89). The anti-inflammatory, antioxidant, antibacterial, anti-caries, and antiplaque properties of propolis support its use in preventing and treating a variety of oral disorders, including dental plaque, gingiva, caries, and oral cancer (90, 91). The formation of dental caries is a chronic process in which a chemical interaction occurs between acid-producing tooth-adherent bacteria and fermented carbohydrate, resulting in dental decay. The most popular pathogenic factor is the high consumption of sugars, as a high number of microorganisms, especially *S. mutans*, which is the most predominant carcinogenic factor in caries (92). The anti-caries and antiplaque activities of crude extracts or hexane fractions of Brazilian propolis have been tested *in vitro* and *in vivo*. The suggested mechanism for the anti-caries activity of propolis is related to the antibacterial action against *S. sobrinus* and *S. mutans* and the inhibition of glucosyltransferase (GTF) enzyme (90, 93). Propolis extracts' cariostatic effects are linked to GTF inhibition rather than antibacterial activity (90). For five weeks, the rats were given hexane fraction of propolis topically twice a day. In *in vivo* study, the fraction greatly reduced the incidence of smooth surface caries while maintaining the percentage of *S. sobriuns* biofilms in the animals' plaque. The high fatty acid content of Brazilian propolis comprising palmitic, oleic, and stearic acids, enables it to exhibit highly potent anti-caries activity when incorporated in a rat diet (93).

The other components in propolis, e.g., CAPE and quercetin exhibit anti-caries properties. CAPE has an antibacterial effect on the most prevalent oral bacteria (*S. mutans, S. sobrinus, Actinomyces viscosus*, and *Lactobacillus acidophilus)* and contributes toward a decrease in the formation of biofilms, as well as reducing their thickness. The antibacterial effect of CAPE is mostly observed toward *S. mutans*. It has been suggested that CAPE penetrates the thick extracellular matrix biofilm and damages the cells, increasing the number of dead bacteria. When administered at half of its minimum inhibitory concentration (MIC), CAPE has been shown to reduce the acidity of *S. mutans* at pH 5, resulting in cell death (94). Quercetin in the Ardabil EEP with a MIC value in the range of 3.12–100 μg/mL was shown to inhibit the growth of oral streptococci and reduce biofilm formation (95). Topical treatment of red propolis (800 μg/mL) in the form of neovestitol–vestitol has been shown to prevent the formation of biofilm and reduce the bacteria-derived exopolysaccharide (EPS)-rich matrix building of biofilm. The proposed mechanism of action for this is via the inhibition of the GTF-derived EPS enzyme activity, which contributes toward the development of EPS by producing soluble glucan or suppressing certain genes linked with the stress and survival of *S. mutans* (96). Topical application of an EEP varnish (15% propolis) or its bioactive fraction twice a day for 4–5 weeks on rat teeth has been shown to exhibit anti-caries activity comparable to that of the drug conventionally used to treat caries, GS/Duraphat^®^. Also, no toxic effects on fibroblast or osteoblast cells were observed (90).

#### Clinical Studies

The protective and treatment effects of propolis as anti-caries, anti-dental plaques, and anti-gingivitis in periodontal diseases have been detected in various clinical studies (Table 3). Propolis or extract reduces dental biofilm formation, raises salivary pH, reduces plaque formation, and protects the oral cavity from bacterial infection (97, 98, 103).

**Table 3:** The potency of propolis against periodontal diseases clinically.

**Condition or disease**	**Recruitment status**	**Bee product/form/Doses**	**Inclusion criteria**	**Type of the study/Clinical trials phase/No of participants**	**Mode of action**	**References**
Dental plaque	Completed	Propolis/Tablet/Propolis tablet, twice a day, for 7 days, giving a 30 day interval between the test and the control tablet.	Age: 10–19 years/Eligible gender: Male and female/ Healthy volunteer without any caries	Interventional/Phase 2, 3/30 P	Causes decline of dental biofilm. Increasing the salivary pH value.	(97)
Dental plaque Gingivitis	Completed	Green propolis/Mouthwash (MGP 5%)/MGP 10 mL, twice a day, for 90 days	Eligible age: 18–60 years Eligible gender: Male and female	Interventional/Phase 2/25 P	Enhances teeth health. Reducing the number of plaques and gingivitis. Prevent infection caused by bacteria in the oral cavity.	(98)
Dental pulp necrosis Periapical abscess Root canal infection	Completed	Ethanolic extract of propolis (EEP) mixed with ciprofloxacin or metronidazole powder in the ratio (1:1)/Antibiotic paste/antibiotic paste was injected in canals by using a sterile plastic syringe, for 18 months	Eligible age: 8 to 18 years Eligible gender: Male and female Patients with: Previous trauma and the anterior teeth with the immature apex	Interventional/Phase 4/40 P	Improvement in root formation. Slight difference in root thickness and length.	(99)
Caries dental caries risk	Completed	Chlorhexidine (12%), propolis (1%) and clove oil (1%)/Mouthwash/Propolis mouthwash, divided into 2 groups as T1 one time per day and T2 twice per day, for one week every month, for 6 months	Eligible age: 8 to 18 years Eligible gender: Male and female Patient with severe risk for caries	Interventional/phase/Not applicable/64 P	Not reported	(100)
Caries risk	Unknown	Propolis/Tooth paste/twice a day, for 6 months	Eligible age: > 18 years Eligible gender: Male and female Patient with high risk for caries. Not take any medicine no interfere with salvia	Interventional, Phase not applicable/40 P	Not reported	(101)
Early childhood caries	Completed	Propolis/Chewing gum propolis/Mouthwash/Propolis 2%, twice daily, for 2 weeks	Eligible age: 6 to 8 years Eligible gender: Male and female Patients with high risk for caries and not having any systematic condition	Interventional/Phase 2/60 P	Not reported	(102)
Streptococcal infections Saliva altered	Completed	Propolis/Varnish/Green propolis 15%, for 30 days after the application of propolis varnish	Eligible age: 8 to 10 years/Eligible gender: Male and female For 3 weeks before the experiment. The patient who do not use any mouthwash or drug especially antibiotics	Interventional/Phase 1, 2/11 P	Inhibition of microorganisms of oral cavity especially *Streptococcus mutans*. Inhibition of biofilm formation.	(103)
Dentin caries	Completed	Propolis/Extract/Propolis, time treatment for first group was one month) and second group was 3 months.	Eligible age: 18 to 80 years Eligible gender: Male and female	Interventional/Phase not applicable/80 P	Not reported	(104)
Chronic periodontitis	Unknown	Propolis	Eligible age: 25 to 50 years Eligible gender: Male and female	Interventional/Phase not applicable/20 P	Not reported	(105)
Periodontitis	Unknown	Alcohol-free mouthwash containing 5% green propolis	Eligible age: 18 to 60 years Eligible gender: Male and female	Interventional/Phase 3/2 P	Not reported	(106)

### Skin Diseases

#### Preclinical Studies

##### Melanogenesis

Melanin pigmentation determines the color of animals' and humans' skin. Melanin protects the skin from UV rays, excessive accumulation of melanin can lead to major skin problems such discoloration, nevus, melasma, and skin aging (107). In a mouse melanocyte cell line (B16F1), royal jelly has exhibited anti-melanogenesis activity, as it decreases mRNA transcription and thus the expression of the tyrosinase enzyme, leading to a decrease in melanin content (108). 10-HDA has shown anti-pigmentation activity *in vivo* using the animal model. 10-HDA decreases tyrosinase, tyrosinase-related protein (TRP)-1, and TRP-2 mRNA transcription by suppressing the microphthalmia-associated transcription factor in B16F10 melanoma cells (109).

##### Wound Healing

Skin is the main physical barrier of the body, which protects it from harmful environmental agents. Bee products have long been used in skincare and are present in many dermatological preparations for purposes, such as skin refreshing, skin whitening, the treatment of burns, and wound healing (110). Royal jelly has antimicrobial activity and enhances the healing of damaged tissues (111). Water-soluble RJPHs, such as MRJP2, MRJP3, and MRJP7, induce the proliferation, differentiation, and migration of human epidermal keratinocyte cells to promote wound closure (112). Royal jelly increases collagen formation and shows anti-inflammatory properties (113). 10-HDA, defensin-1, and MRJP3, which are components of royal jelly, have an inhibitory effect on pro-inflammatory cytokines, such as IL-1 and IL-6, without harming the macrophages themselves. MRJP induces the proliferation and migration of specific cells, such as fibroblasts.

Propolis' anti-inflammatory, antioxidant, and antibacterial properties may have contributed to its efficacy in wound healing (114, 115). Propolis reduces the concentration of pro-inflammatory proteins, such as cyclooxygenase (COX-1 and COX-2), proteinase, and prostaglandins (prostaglandin E) owing to the presence of flavonoid and phenolic compounds, such as CAPE (116, 117). In addition, the topical application of propolis on diabetic wounds has reduced/declined IL-1β, IL-6, TNF-α, and MMP9 levels in STZ-induced type I diabetic mice (35). Propolis has also enhanced wound healing in rats and reduced the closure time by stimulating collagen formation and keratinocyte migration. The antibacterial effect of a mixture of two different types of propolis collected from different regions in Iraq has been tested on *Escherichia coli, Staphylococcus aureus*, and *Candida albicans*. A combination of the two types of propolis was found to significantly improve the anti-inflammatory, antioxidant, and re-epithelization properties of the materials compared with their use alone, leading to an acceleration in the wound healing process (118).

Tissue engineering is a scientific technique that focuses on remolding tissue damage and organ substitutes (119). In tissue engineering, different functional polymer membranes, such polyvinyl alcohol (PVA), and cellulose acetate are used to fabricate nanostructure-based scaffolds for use in wound dressings. *In vivo* and *in vitro* studies have shown that the combination of propolis with the polymer wound dressings helps to promote their usability in the wound healing process (120–122). The loading of propolis in a PVA nanofiber wound dressing has promoted wound closure of up to 68% after seven days of treatment in a diabetic wound healing model (121). The treatment of male rats suffering from second-degree burns with propolis associated in natural rubber membrane has resulted in regeneration, re-epithelization, collagen deposition, and a decrease in the number of inflammatory cells in the animals (123). Moreover, bilayer wound dressing was prepared using a polycaprolactone/gelatin scaffold (sub-layer) that was electrospun on a dense membrane consisting of an EEP membrane (top layer). The bilayer was then shown to induce wound healing and reduce the closure time. In general, the bilayer technique improves the biocompatibility, biodegradability, and mechanical proprieties of dressings in wound healing. The presence of propolis in the first layer provides protection for wounds against infections due to its antibacterial and antifungal properties (124).

## Clinical Studies of Bee Products Against Wounds

Honey's antibacterial activity, immunological modulatory capabilities, and biocompatibility all contribute to its usefulness as a wound-healing agent (125). Honey in combination with other therapies has superior antibacterial action, suggesting that it may be useful for malignant wounds, diabetic foot ulcers, wounds, leg ulcers, and burn wounds (Table 4) (126, 127, 133, 137, 139). PedyPhar^®^ Ointment consists of royal jelly and panthenol enhanced the healing rate in patients with diabetic foot ulcers. As well, a daily application of Leptospermum honey gel on ulcers or wounds promoted tissue proliferation and decreased the accumulation of necrotic tissue in wounds (Table 4) (128).

**Table 4:** The potency of some bee products against wounds clinically.

**Condition or Disease**	**Recruitment status**	**Bee product/Form/Doses**	**Inclusion Criteria**	**Type of the study/ Clinical trials phase/No of participants**	**Mode of action**	**References**
Malignant wounds	Completed	Honey product/alginate wound dressing/Honey product dressing/Not reported	Eligible age: > 18 years Eligible gender: Male and female Patient with cancer wounds > 2 cm/ Patients take anti-neoplasm cure	Interventional/Phase 3/70 P	Enhances wound healing by reducing the size, pain and healing of wounds	(126)
Diabetic foot ulcer (T2DM)	Completed	Honey/gel sheet wound dressing/Honey gel sheet, applied on ulcer daily, for 12 weeks	Eligible age: 40 to 85 years Eligible gender: Male and female Ulcer size: ≥ 1 cm Ulcer without or with mild infection. Ulcers should be at or below malleolar region of the foot. Patients with no expected surgery within 12 weeks of administration	Interventional/Phase, not applicable/31 P	Reducing ulcer size. Decreasing level of wound fluid concentration of MMP9, TNF-α and IL-1α among groups, *P* < 0.05 for all tests.	(127)
Diabetic foot ulcer	Terminated	Royal jelly and panthenol (PedyPhar^®^ Ointment)/PedyPhar, 3–5 mm was distributed on a dressing and then apply the dressing to the ulcer until the complete healing.	Eligible age: 18 to 75 years Eligible gender: Male and female/ Type 1 or type 2 diabetes mellitus with diabetic foot ulcer	Interventional/Phase 3/47 P	Reduction of the necrotic tissue resulted in enhancement of healing rate.	(128)
Wounds ulcer	Terminated	Leptospermum honey (Medi honey^®^)/Gel/Medihoney applied daily, for 90 days	Eligible age: > 18 years Eligible gender: Male and female Necrotic tissue ≥ 50 % in wound and surface area > 1 cm^2^ to <64 cm^2^ For diabetes HbA1c <12.0%. Pre-albumin > 16 mg/dl within the period of study	Interventional/Phase, not applicable/10 P	Reduction of necrotic tissue in wounds. Promoting the tissue proliferation.	(129)
Diabetes mellitus amputation wound and toe wound	Recruiting	Honey/MelectisG wound dressing/Honey Melectis G [honey (99.8%) and hyaluronic acid (0.2%)], until complete healing (12 months maximum).	Eligible age: > 18 years Eligible gender: Male and female Diabetic patients who have one or more amputated toes, four days before inclusion in the study. Patients to write the consent form.	Interventional/Phase, not applicable/50 P	Not reported	(130)
Palatal wound healing	Completed	0.05 cm^3^ of honey per 1cm of laceration, given every predetermined wound care schedule	Eligible age 10–60 years Eligible gender: Male and female	Interventional/Phase, 3/35 P	Not reported	(131)
Diabetic wound	Not yet recruiting	Honey	Eligible age: 18–70 years Eligible gender: Male and female	Interventional/Phase, not applicable/76 P	Wound size reduction.	(132)
Wound healing	Completed	Honey	Eligible age: 18–100 years Eligible gender: Male and female	Interventional/Phase, not applicable/40 P	Within 6 months after vascular surgery, the healing rate of acute or chronic surgical wounds.	(133)
Necrotizing fascitis wounds	Recruiting	Topical honey to be used for dressing 4 mL per square inch.	Eligible age: 18–70 years Eligible gender: Male and female	Interventional/ Phase, not applicable/ 326 P	Early wound healing. Decreased hospital stay.	(134)
Wound healing	Completed	ziziphus honey	Eligible age: 18–40 years Eligible gender: Male and female	Interventional/Phase, 3/30 P	Not reported	(135)
Pressure ulcer	Recruiting	Aloe and propolis applied	Eligible age: 18 Years and older Eligible gender: Male and female	Interventional/Phase, not applicable/66 P	Not reported	(136)
Burn wound	Completed	Propolis	Eligible age: 8 to 12 Weeks Eligible gender: Male and female	Interventional/ Phase, not applicable/36 P	Not reported	(137)
Wound healing	Recruiting	New Mexico Honey	Eligible age: 16-75 years Eligible gender: Male and female	Interventional/Phase, 2/ 60 P	Not reported	(138)
Leg ulcer	Completed	Honey and ionic silver dressing	Eligible age: 18 Years and older Eligible gender: Male and female	Interventional/Phase, 2/30 P	Granulation and/or epithelial tissue progression as observed	(139)
Wound healing	Recruiting	Novel biomaterial containing gelatin, manuka honey, and hydroxyapatite	Eligible age: 18 Years and older Eligible gender: Male and female	Interventional/Phase, not applicable/80 P	Not reported	(140)
Split-skin grafted third-degree burn wound	Recruiting	Manuka-Honey	Eligible age: 18 Years and older Eligible gender: Male and female	Interventional/Phase, not applicable/20 P	Not reported	(141)

### Peptic Ulcers

#### Preclinical Studies

Vomiting, nausea, postprandial abdominal pain, and weight loss are typical symptoms associated with peptic ulcer. The term “peptic ulcer” refers to the irritation of the digestive tract, mostly of the stomach or proximal duodenum wall because of the hyper secretion of peptic acid. The use of non-steroidal anti-inflammatory drugs (NSAIDs) or aspirin, and *Helicobacter pylori* infections could all lead to peptic ulcers (5). *H. pylori* are Gram-negative bacteria that infect the human mucosa. *H. pylori* as a causative factor in gastric inflammatory, ulcers, and cancers stimulate the expression of the outer membrane proteins related to epithelial gastric cells (142). Bacterial honey, *Bacillus subtilis*, is a source of different types of levans. *Bacillus sp*. levan (200 mg/kg) has been shown to act as a probiotic agent against gastric ulcers induced by ethanol in adult male Sprague–Dawley rats. The probiotic activity of levan enables it to cover the stomach epithelial cells and prevent the adhesion of *H. pylori* (143). The suggested mechanism of the gastro-protective effects of propolis and its isolated metabolites drupanin, artepillin C, kaempferide, and aromadendrin-4-*O*-methyl-ether are mostly associated with their anti-inflammatory, antioxidant, and inhibitory properties toward gastric secretion (144).

The administration of hydroalcoholic extracts of red propolis (HERP, 250 and 500 mg/kg) or its formononetin isoflavonoid (10 mg/kg) has provided protection against ulcer formation, decreased the volume of gastric secretions, and led to healing the damaged areas of the mucosa in a dose-dependent manner. HERP has been shown to inhibit *H. pylori* infection and decrease the diameter of the affected zone by 13.0 ± 2.0 mm at 100 mg/mL (145). The MAPK and NF-κB pathways promote the pathogenic action of *H. pylori*. An ethanolic extract of Korean propolis has reduced levels of pro-inflammatory interleukins, such as IL-12, IL-8, IL-1β, and COX-2 via the inhibition of the MAPK and NF-κB pathways (146). KAE (160 mg/kg) causes a decrease of TNF-α by 48% and IL-1β by 37% in the gastric ulcer. It also increases the level of NO vasodilator factor and increasing the blood flow to the gastric mucosa (147).

**Figure 5:**
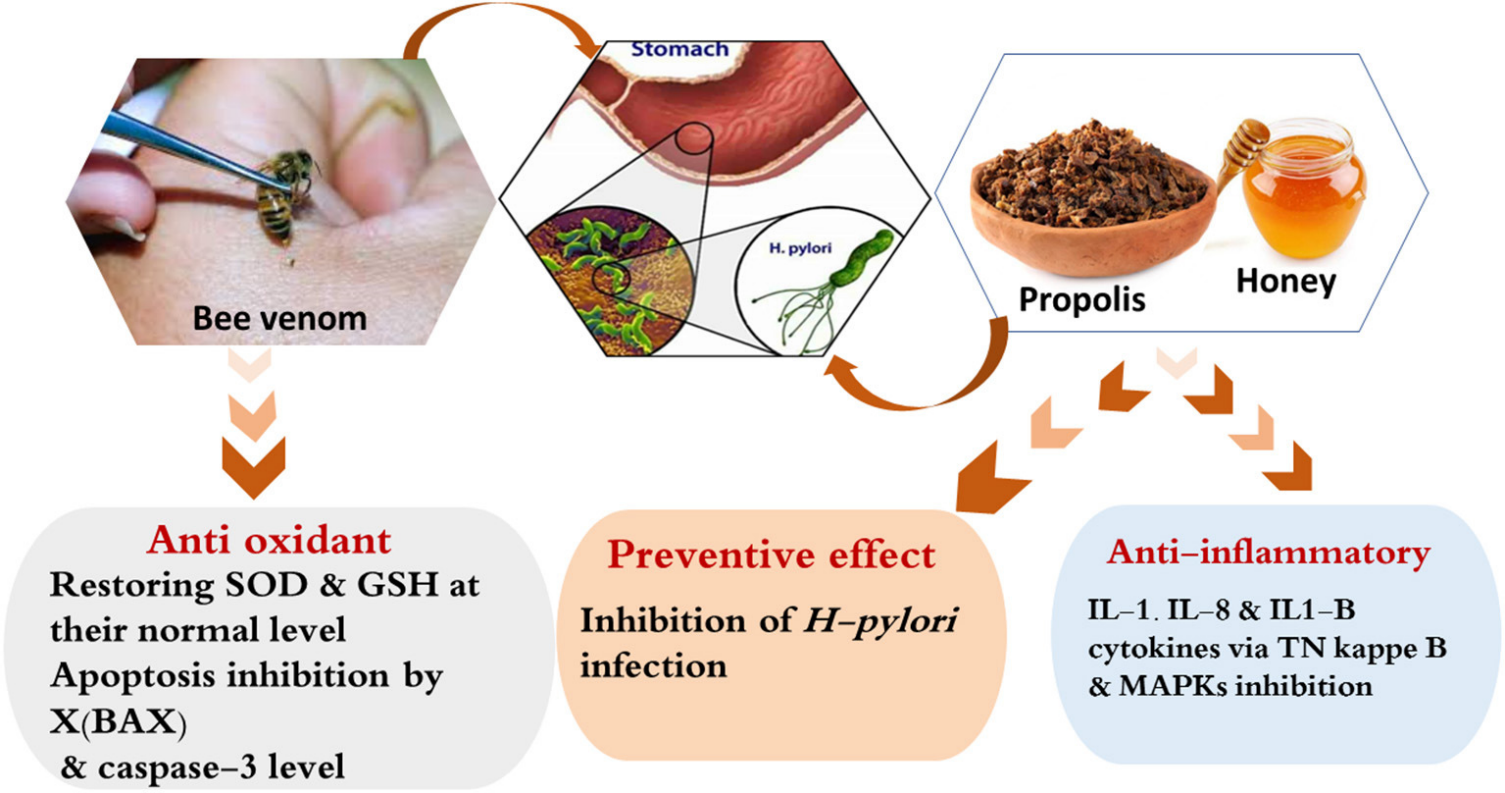
Possible mechanism of actions explaining the anti-peptic ulcers effect of the bee products.

Bee venom has been shown to exhibit anti-inflammatory and anti-apoptotic properties and have a protective effect against NSAID-induced gastric ulcers. An intraperitoneal injection of 2 mg/kg of bee venom into Sprague–Dawley rats with ASA-induced peptic ulcers has been shown to reduce the levels of pro-inflammatory cytokines, such as TNF-α and maintain normal levels of antioxidant enzymes, such as SOD and GSH. In addition, it decreases the expression of caspase-3 and B-cell lymphoma associated X, and enhances the recovery of mucosal damage and inhibiting apoptosis in gastric cells (148). Propolis, honey, and bee venom mostly demonstrate protective and healing effects on peptic ulcers because of their anti-inflammatory, antioxidant, and antimicrobial properties (Figure 5).

### The Safety of Bee Products

An allergic reaction could occur following the use of bee products but it is rare, hypersensitive reactions to honey vary from a cough to anaphylaxis (28, 149, 150). This allergic reaction could be attributed to various causes, such as the bee pollen content, mold spores, algae, and other bee-derived proteins (151). The consumption of royal jelly could be associated with serious adverse reactions, such as contact dermatitis, acute asthma, anaphylaxis, and even death for some individuals (152, 153). In countries with a high utilization of royal jelly, positive skin prick tests are used to diagnose atopic reactions (154). Clinical studies have shown that serum IgE antibodies against a number of royal jelly components are found in subjects with clinical sensitivity. These IgE anti-binding proteins are mostly derived from major RJPHs, especially MRJP1 and MRJP2. Antibody-binding proteins with a molecular weight of 25–55 kDa have been identified in a study in the sera of subjects that showed an adverse reaction to royal jelly, where two proteins with molecular weights of 47 and 55 kDa may be the most diagnostic biomarkers discovered so far (155, 156). IgE-binding proteins are not specific to royal jelly and can be present in other cases of allergies. This can be explained by the contamination and cross-reactivity of RJPHs with inhalant allergens, such as pollen.

Airborne pollen is a known cause of respiratory allergy, which might later progress to a life-threatening allergic reaction. Although bee pollen is mainly made up of the pollen of entomophilous plants that attract insects for their pollination, it also contains a significant amount of pollen from wind-pollinated trees or weeds (anemophilous plants), which may induce hypersensitive reactions after the ingestion of bee pollen (28, 156). In addition, bee pollen can be contaminated by different types of fungi, such as *Aspergillus, Cladosporium*, and *Alternaria*, which can induce allergic reactions (28).

Because the Gram-positive Bacterium *Clostridium botulinum*, which is considered a potential risk factor for baby botulism, and can be found in both honey and bee pollen, the World Health Organization prohibits the usage of honey for newborns under the age of one. *C. botulinum's* can produce eight distinct toxins (A, B, C1, C2, D, E, F, G) (157–159). Also, allergic reactions to bee venom have been observed. As a result of this hypersensitivity, anaphylaxis may arise and develop into fatal shock (160). Compared with airborne allergens, such as mites and pollen (161), venom allergens are directly injected into the skin and easily reach the bloodstream. This promotes an immune response that manifests in the form of edema, erythema, and pruritus at 10 cm in diameter or greater. The skin inflammation that occurs in response to venom allergens takes 1–2 days to appear and 3–10 days to be resolved.

Therefore, the accurate diagnosis of an allergy to insect venom is important and is based on history, skin tests, and the determination of specific IgE antibodies for bee venom (162, 163). The allergen crosslinking of IgE bound to mast cells leads to the release of histamine, lipid mediators, enzymes, cytokines, and chemokines responsible for the acute allergic response. Cross-reactivity can occur due to similarities between the composition of venom and the structures of single allergens (164). Similarities can be found between different bee species, and even between bees and wasps (165). Cross-reactive carbohydrate determinants were found to act as antigens against IgE in the sera of patients with bee venom allergies, so this may be the cause of honeybee venom allergies (165).

The main therapeutic options for bee venom allergy are in the form of emergency medications, including antihistamines, epinephrine (adrenaline) injection, and corticosteroids. Moreover, venom-specific immunotherapy has been developed, whereby repeated injections of increasing venom doses are given to a patient over a period of years to reduce allergic symptoms (160, 166, 167).

## Conclusion

Despite the vital role of inflammation as a potential defense mechanism of the body, its uncontrolled regulation can result in various autoimmune diseases, which cause serious health problems. Bee products are apitherapeutic agents, rich in hundreds of bioactive chemical compounds that account for their various biological activities, such as their antioxidant, anti-inflammatory, immunomodulatory, neuroprotective, and antimicrobial properties. Different bee products and their bioactive ingredients have been shown to be successful in the protection and treatment of various autoimmune diseases in both preclinical and clinical studies.

In preclinical studies, bee products improve the immune response via the modulation of B and T lymphocytes function and chemotaxis. Also, they inhibit the production of inflammatory cytokines and regulate intracellular signaling pathways. Taken together, these results supported the clinical studies of bee products. For instance, in diabetes, propolis helped increasing TLR-2 and TLR-4, thus activating the B and T function, improving the lipid profile, reducing the ROS and modulating the HbA1C and FSG glycemic factors. Meanwhile, honey, propolis and their phenolic compounds can act as chemoprevention and tumor growth inhibitors in cancer cells. Antibacterial, anti-caries, and antiplaque properties of propolis play important role in the treatment of periodontal diseases such as gingival and caries. The gastroprotective effect of honey and propolis is related to the inhibition of *H-pylori* infection and the reduction of the NSAIDs-related inflammation.

However, to date, there have been a few applicable clinical studies on the roles that bee products play in the treatment of inflammatory and autoimmune diseases. Also, adverse allergic reactions and unselective toxicity still present challenges in the development of bee products if they are to be used to achieve the greatest scientific and commercial outcomes in drug discovery development. Therefore, it is important to carry out more clinical studies to explore the benefits of the use of bee products in future drug discovery. It is also critical to take care of honeybees as a source of bee products, especially in the aftermath of colony collapse disorder.

## Author Contributions

HE-S and SK: conceptualization and supervision. NE, SK, and AAA: writing of the original draft. SK, HE-S, MR, HA, AFA, CZ, YA, SA, HT, BX, and KW: revision and editing of the final version. All authors have read and agreed to the published version of the manuscript.

## Funding

This work was supported by Swedish Research links (Grant VR 2016–05885), National Natural Science Foundation of China (Grant No. 32172791), and State Key Laboratory of Animal Nutrition (2004DA125184F1904).

## Conflict of Interest

The authors declare that the research was conducted in the absence of any commercial or financial relationships that could be construed as a potential conflict of interest.

## Publisher's Note

All claims expressed in this article are solely those of the authors and do not necessarily represent those of their affiliated organizations, or those of the publisher, the editors and the reviewers. Any product that may be evaluated in this article, or claim that may be made by its manufacturer, is not guaranteed or endorsed by the publisher.
